# A unified masking approach

**DOI:** 10.1007/s13389-018-0184-y

**Published:** 2018-03-15

**Authors:** Hannes Gross, Stefan Mangard

**Affiliations:** Graz University of Technology, Inffeldgasse 16a, 8010 Graz, Austria

**Keywords:** Masking, Hardware security, Threshold implementations, Domain-oriented masking, Side-channel analysis

## Abstract

The continually growing number of security-related autonomous devices requires efficient mechanisms to counteract low-cost side-channel analysis (SCA) attacks. Masking provides high SCA resistance at an adjustable level of security. A high level of resistance, however, goes hand in hand with an increasing demand for fresh randomness which drastically increases the implementation costs. Since hardware-based masking schemes have other security requirements than software masking schemes, the research in these two fields has been conducted quite independently over the last 10 years. One important practical difference is that recently published software schemes achieve a lower randomness footprint than hardware masking schemes. In this work we combine existing software and hardware masking schemes into a unified masking algorithm. We demonstrate how to protect software and hardware implementations using the same masking algorithm, and for lower randomness costs than the separate schemes. Especially for hardware implementations, the randomness costs can in some cases be halved over the state of the art. Theoretical considerations as well as practical implementation results are then used for a comparison with existing schemes from different perspectives and at different levels of security.

## Introduction

One of the most popular countermeasures against side-channel analysis attacks is Boolean masking. Masking is used to protect software implementations as well as hardware implementations. However, since it was shown that software-based masking schemes (that lack resistance to glitches) are in general not readily suitable to protect hardware implementations [18], the research has split into masking for software implementations and masking for hardware implementations.

The implementation costs of every masking scheme is thereby highly influenced by two factors: at first, the number of shares (or masks) that are required to achieve *d*th-order security, and second the randomness costs for the evaluation of nonlinear functions. For the first one, there exists a natural lower bound of $d+1$ shares in which every critical information needs to be split in order to achieve *d*th-order security.

For the evaluation of nonlinear functions, the number of required fresh random bits has a huge influence on the implementation costs of the masking because the generation of fresh randomness requires additional chip area, power and energy, and also limits the maximum throughput. Recently proposed software-based masking schemes require (with an asymptotic bound of $d(d+1)/4$) almost half the randomness of current hardware-based masking schemes.

*Masking in hardware* With the threshold implementations (TI) scheme by Nikova et al. [19], the first provably secure masking scheme suitable for hardware designs (and therefore resistant to glitches) was introduced in 2006. TI was later on extended to higher-order security by Bilgin et al. [3]. However, the drawback in the original design of TI is that it requires at least $t d +1$ shares to achieve *d*th-order (univariate [20]) security where *t* is the degree of the function. In 2015, Reparaz et al. [21] demonstrated that *d*th-order security can be also achieved with only $d+1$ shares in hardware. A proof-of-concept was presented at CHES 2016 by De Cnudde et al. [6] requiring $(d+1)^2$ fresh randomness. Gross et al. [12, 13] introduced the so-called domain-oriented masking (DOM) scheme that lowers the randomness costs to $d (d+1)/2$.

*Masking in software* Secure masked software implementations with $d+1$ shares exist all along [7, 16, 22]. However, minimizing the requirements for fresh randomness is still a demanding problem that continues to be researched. Since efficient implementation of masking requires decomposition of complex nonlinear functions into simpler functions, the reduction in randomness is usually studied on shared multiplications with just two shared input bits without a loss of generality.

In 2016, Belaïd et al. [2] proved an upper bound for the randomness requirements of masked multiplication of $O(d \log d)$ for large enough *d*’s, and a lower bound to be $d + 1$ for $d \le 3$ (and *d* for the cases $d \le 2$). Furthermore, for the orders two to four, Belaïd et al. showed optimized algorithms that reach this lower bound and also introduced a generic construction that requires $\frac{d^2}{4} + d$ fresh random bits (rounded). Recently, Barthe *et al.* [1] introduced a generic algorithm that requires $\lceil \frac{d}{4} \rceil (d+1)$ fresh random bits. Barthe et al.’s algorithm saves randomness in three of four cases over Belaïd et al.’s algorithm, but for the remaining cases it requires one bit more.

Please note, even though Barthe *et al. * states that their parallelization consideration makes their algorithm more suitable for hardware designs, it stays unclear how these randomness optimized multiplication algorithms can be securely and efficiently implemented in hardware with regard to glitches.

*Our contribution* In this work, we combine the most recent masking approaches from both software and hardware in a unified masking approach (UMA). The basis of the generic UMA algorithm is the algorithm of Barthe *et al. * which we combine with DOM [13]. The randomness requirements of UMA are in all cases less or equal to generic software masking approaches. As a non-generic optimization, for the second protection order, we also take the solution of Belaïd *et al. * into account.

We then show how the UMA algorithm can be efficiently ported to hardware and thereby reduce the asymptotic randomness costs to $d(d+1)/4$. Therefore, we analyze the parts of the algorithm that are susceptible to glitches and split the algorithm into smaller independent hardware modules that can be calculated in parallel. As a result, the latency in hardware is at most five cycles.

Finally, we compare the implementation costs and randomness requirements of UMA to the costs of DOM in a practical and scalable case study for protection orders up to 15. We round out this work with a thorough empirical and formal analysis of the SCA resistance for the UMA case study.

*This paper is organized as follows* In Sect. 2, we give a short introduction to randomness efficient Boolean masking in software and hardware, and introduce the existing masked multiplication algorithms that we use in Sect. 3 to design UMA in software. The secure implementation of UMA in hardware is then discussed in Sect. 4. In Sect. 5, we introduce a case study in which we compare UMA and DOM on the basis of a generic and configurable hardware implementation of the authenticated encryption design Ascon. The side-channel security of the UMA S-box design is then at first analyzed empirically using statistical *t* tests on simulated power traces in Sect. 6 before we strengthen the analysis with a formal verification in Sect. 7. We conclude this paper in Sect. 8 with a discussion on the suitability of UMA when the generation of randomness is taken into account.

## Boolean masked multiplication

The main problem in designing Boolean masked circuits is the secure and efficient implementation of nonlinear functions. Because the costs for the secure implementation of functions with higher algebraic degree grow exponentially, complex functions are usually broken down into functions of lower degree. The problem of designing Boolean masked circuits can therefore be narrowed down to the efficient construction of masked multiplications of two elements over a finite field. However, even when we look at one-bit multiplications in the following for convenience reasons, all statements are applicable to multiplications in arbitrary finite fields in a straightforward manner.

We use similar notations as Barthe et al. [1] to write the multiplication of two variables *a* and *b*. In shared form, the multiplication of $\varvec{a} \cdot \varvec{b}$ is given by Equation 1 where the elements of the vectors $\varvec{a}$ and $\varvec{b}$ are referred to as the randomly generated sharing of the corresponding variable. For any possible randomized sharing, the equations $a = \sum _{i=0}^{d}{a_i}$ and $b = \sum _{j=0}^{d}{b_j}$ always need to be fulfilled, where $a_i$ and $b_j$ refer to individual shares of $\varvec{a}$ and $\varvec{b}$, respectively.

1 $$\begin{aligned} \varvec{q} = \varvec{a} \cdot \varvec{b} = \sum _{i=0}^{d} \sum _{j=0}^{d} a_i b_j \end{aligned}$$

In order to correctly implement this multiplication in shared form, Equation 1 needs to be securely evaluated. In particular, summing up the multiplication terms $a_i b_j$ needs to result again in a correct sharing of the result *q* with $d+1$ shares, and needs to be performed in such a way that an attacker does not gain any information on the unshared variables *a*, *b*, or *q*. To achieve *d*th-order security, an attacker with the ability to “probe” up to *d* signals during any time of the evaluation should not gain an advantage in guessing any of the multiplication variables. This model is often referred to as the so-called (*d*-)probing model of Ishai et al. [16] which is linked to differential side-channel analysis (DPA) attacks over the statistical moment that needs to be estimated by an attacker for a limited set of noisy power traces [9, 22]. This task gets exponentially harder with increasing protection order *d* if the implementation is secure in the *d*-probing model [5].

However, directly summing up the terms $a_i b_j$ does not even achieve first-order security regardless of the choice for *d*. To make the addition of the terms secure, fresh random shares denoted as *r* in the following are required that are applied to the multiplication terms on beforehand. The number of required fresh random bits and the way and order in which they are used is essential for the correctness, security, and efficiency of the shared multiplication algorithm.

*DOM multiplication algorithm* As a starting point of our randomness considerations we use the multiplication matrix of the DOM scheme [14] which was designed to protect hardware implementations. For the hardware implementation, the parentheses indicate registers at the according positions. For the moment, however, we do not consider registers but instead assume a software implementation for which all stated equations are evaluated from left to right and with respect to the parentheses. An example is given in Equation 2.

2 $$\begin{aligned} q_0&= a_0 b_0 + (a_0 b_1 + r_0) + (a_0 b_2 + r_1) + (a_0 b_3 + r_3) \dots \nonumber \\ q_1&= (a_1 b_0 + r_0) + a_1 b_1 + (a_1 b_2 + r_2) + (a_1 b_3 + r_4) \dots \nonumber \\ q_2&= (a_2 b_0 + r_1) + (a_2 b_1 + r_2) + a_2 b_2 + (a_2 b_3 + r_5)\dots \nonumber \\ q_3&= (a_3 b_0 + r_3) + (a_3 b_1 + r_4) + (a_3 b_2 + r_5) + a_3 b_3 \dots \nonumber \\&\dots \end{aligned}$$

This shared multiplication requires $d(d+1)/2$ fresh random bits which results from the fact that multiplication terms where $\varvec{a}$ and $\varvec{b}$ have the same index (inner-domain terms) do not require fresh randomness, and for the remaining cross-domain terms the same random bit is used as for terms with mirrored indices. For example, the term $a_0 b_1$ uses the same random bit $r_0$ for remasking as $a_1 b_0$. The correctness of the shared multiplication thus results from the fact that it contains all terms resulting from Equation 1 and all fresh random *r* bits appear twice which therefore cancel each other out when the sum over all $\varvec{q}$ shares is calculated. The DOM algorithm is especially suited for hardware implementations because it does not require control over the order in which the (remasked) multiplications terms are summed up in each domain, which would require additional registers. However, by introducing a better control over the order in which these terms are summed up (more registers or using software implementations) the randomness requirement can be lowered as *e.g. * the algorithm of Barthe *et al. * demonstrates.

*Barthe et al.’s algorithm* The essence of Barthe et al.’s algorithm is that by grouping the multiplication terms and the used random bits in a certain way, the randomness can be securely reused and in a more efficient manner than in Equation 2. Please note that for the moment we just consider the original software implementation of the algorithm for which all stated equations are evaluated from left to right. Parentheses indicating registers are thus omitted.

Instead of using one random bit to protect two mirrored terms ($a_i b_j$ and $a_i b_j$, where $i\ne j$) as in Equation 2, the same fresh random bit can be used again to protect another pair of mirrored terms. The multiplication matrix for the shared $\varvec{q}$ ($=\{q_0, q_1,\dots , q_d\}$) can thus be written according to Equation 3 for $d=4$ as an example. Here the random bit $r_0$ is used to secure the absorption of the terms $a_0 b_1$ and $a_1 b_0$ in $q_0$ as well as for the terms $a_4 b_1$ and $a_1 b_4$ in $q_4$.

3 $$\begin{aligned} q_0&= a_0 b_0 + r_0 + a_0 b_1 + a_1 b_0 + r_1 + a_0 b_2 + a_2 b_0 \nonumber \\ q_1&= a_1 b_1 + r_1 + a_1 b_2 + a_2 b_1 + r_2 + a_1 b_3 + a_3 b_1 \nonumber \\ q_2&= a_2 b_2 + r_2 + a_2 b_3 + a_3 b_2 + r_3 + a_2 b_4 + a_4 b_2 \nonumber \\ q_3&= a_3 b_3 + r_3 + a_3 b_4 + a_4 b_3 + r_4 + a_3 b_0 + a_0 b_3 \nonumber \\ q_4&= a_4 b_4 + r_4 + a_4 b_0 + a_0 b_4 + r_0 + a_4 b_1 + a_1 b_4 \end{aligned}$$

A vectorized version of Barthe et al.’s algorithm is given in Equation 4 where all operations are performed share-wise from left to right. Accordingly, the vector multiplication is the multiplication of the shares with the same share index, *e.g.* $\varvec{ab}=\{a_0 b_0, a_1 b_1,\dots , a_d b_d\}$. Additions in the subscript indicate an index offset of the vector modulo $d+1$ which equals a rotation of the vector elements inside the vector, *e.g.* $\varvec{a}_{+1} = \{a_1, a_2,\dots , a_0\}$. Superscript indices refer to different and independent randomness vectors with a size of $d+1$ random bits for each vector.

4 $$\begin{aligned} \varvec{q}&= \varvec{a} \varvec{b} + \varvec{r^0} + \varvec{a} \varvec{b}_{+ 1} + \varvec{a}_{+ 1} \varvec{b} + \varvec{r^0}_{+ 1} + \varvec{a} \varvec{b}_{+ 2} + \varvec{a}_{+ 2} \varvec{b} \nonumber \\&\quad +\,\varvec{r^1} + \varvec{a} \varvec{b}_{+ 3} + \varvec{a}_{+ 3} \varvec{b} + \varvec{r^1}_{+ 1} + \varvec{a} \varvec{b}_{+ 4} + \varvec{a}_{+ 4} \varvec{b} \nonumber \\&\quad +\, \varvec{r^2} + \varvec{a} \varvec{b}_{+ 5} + \varvec{a}_{+ 5} \varvec{b} + \varvec{r^2}_{+ 1} + \varvec{a} \varvec{b}_{+ 6} + \varvec{a}_{+ 6} \varvec{b} \dots \end{aligned}$$

At the beginning of the algorithm, the shares of $\varvec{q}$ are initialized with the terms resulting from the share-wise multiplication $\varvec{a b}$. Then there begins a repeating sequence that ends when all multiplication terms are absorbed inside one of the shares of $\varvec{q}$. The first sequence starts with the addition of the random bit vector $\varvec{r^0}$. Then a multiplication term and mirrored term pair ($a_i b_j$ and $a_j b_i$, where $i\ne j$) is added, before the rotated $\varvec{r^0}_{+1}$ vector is added followed by the next pair of terms. The next (up to) four multiplication terms are absorbed using the same sequence but with a new random bit vector $\varvec{r^1}$. This procedure is repeated until all multiplication terms are absorbed. There are thus $\lceil \frac{d}{4} \rceil$ random vectors required with a length of $d+1$ bits each. So, in total the randomness requirement is $\lceil \frac{d}{4} \rceil (d+1)$. In any case, for the last sequence Barthe et al.’s algorithm introduces a new randomness vector that is added once normally and once rotated by one element. This makes the randomness usage of the last sequence less efficient if the last sequence uses less than four multiplication terms per share of $\varvec{q}$. We shall take this up again in Sect. 3 in order to extend Barthe et al.’s algorithm to make it more randomness efficient.

*Randomness bounds* Barthe et al.’s generic algorithm, even though it has the best asymptotic randomness requirement so far, is known to be not optimal in all cases. The work of Belaïd et al. [2] proves a lower bound for the randomness requirement of masked multiplications being $d + 1$ for $d \le 3$ (and *d* for the cases $d \le 2$). This work also introduces a generic masking algorithm along with some brute force searched “optimal” solutions which achieve the stated randomness bound.

**Fig. 1:**
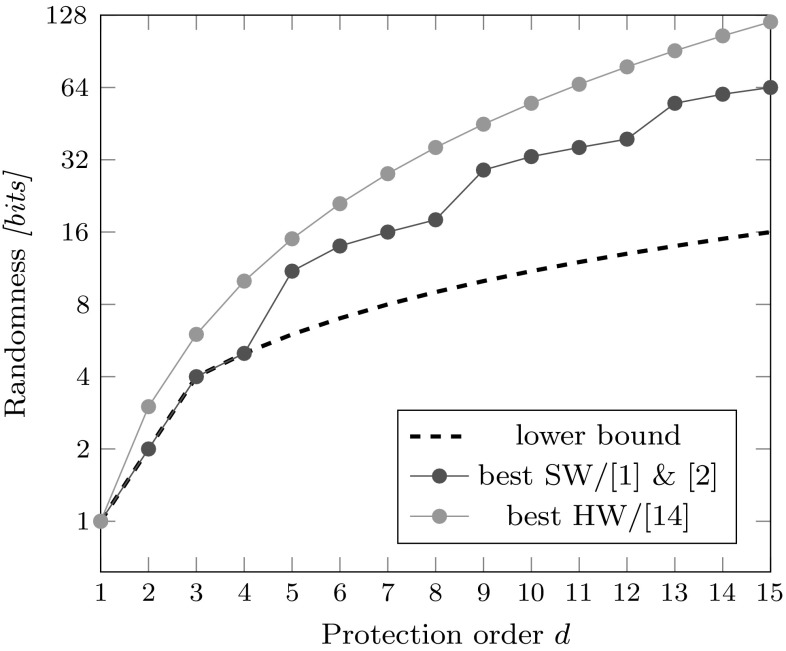
Randomness requirements for the most efficient masked multiplication algorithms reported up to now

Nevertheless, it remains unclear whether or not this stated randomness bound is tight. Furthermore, this lower randomness bound (for $d > 1$) is so far only reached by Belaïd et al.’s brute force searched optimal solutions up to order 4, and in the case of ($d=3$ and $d=4$) by Barthe et al.’s generic algorithm. For protection orders above $d=4$ there is no algorithm known that reaches this lower bound. In addition, Barthe et al.’s algorithm requires one fresh random bit more than Belaïd et al.’s generic algorithm in case $d = 1 \mod 4$.

The randomness requirement is depicted in Figure 1 for which the so far least randomness demanding masked multiplication algorithms in software were merged together, and then compared to the so far least randomness demanding masked multiplication algorithm suitable for hardware implementations (DOM). As it shows, the gap between the state of the art in software- and hardware-based masking is quite significant, and in some cases it is as wide as two times the amount of random bits required for software (for $d = 0 \mod 4$).

In the next sections, we introduce the unified masked multiplication algorithm (UMA) that closes the randomness gap between software-based and hardware-based masking. Furthermore, UMA also lowers the randomness requirement for software for $d = 1 \mod 4$.

## Unified masked multiplication

For the assembly of the unified masked multiplication algorithm (UMA) we extend Barthe et al.’s algorithm with optimizations from Belaïd *et al. * and DOM. We differentiate between four cases for handling the last sequence in Barthe et al.’s algorithm: (1) if the protection order *d* is an integral multiple of 4, we call the last sequence *complete*, (2) if $d \equiv 3 \mod 4$, we call it *pseudo-complete*, (3) if $d \equiv 2 \mod 4$, we call it *half-complete*, and (4) if $d \equiv 1 \mod 4$, we call it *incomplete*. We first introduce each case briefly before giving a full algorithmic description of the whole algorithm.

*(Pseudo-)complete* Complete and pseudo-complete sequences are treated according to Barthe et al.’s algorithm. In contrast to the complete sequence, the pseudo-complete sequence contains only three multiplication terms per share of $\varvec{q}$. See the following example for $d=3$:

$$\begin{aligned} \varvec{q} = \varvec{a} \varvec{b} + \varvec{r^0} + \varvec{a} \varvec{b}_{+ 1} + \varvec{a}_{+ 1} \varvec{b} + \varvec{r^0}_{+ 1} + \varvec{a} \varvec{b}_{+ 2} \end{aligned}$$

*Half-complete* Half-complete sequences contain two multiplication terms per share of $\varvec{q}$. For handling this sequence, we consider two different optimizations. The first optimization requires *d* fresh random bits and is hereafter referred to as Belaïd’s optimization because it is the non-generic solution in [2] for the $d=2$ case. An example for Belaïd’s optimization is given in Equation 5. The trick to save randomness here is to use the accumulated randomness used for the terms in the first functions in order to protect the last function of $\varvec{q}$. It needs to be ensured that $r^0_0$ is added to $r^0_1$ before the terms $a_2 b_0$ and $a_0 b_2$ are added.

5 $$\begin{aligned} q_0&= a_0 b_0 + r^0_0 + a_0 b_1 + a_1 b_0 \nonumber \\ q_1&= a_1 b_1 + r^0_1 + a_1 b_2 + a_2 b_1 \nonumber \\ q_2&= a_2 b_2 + r^0_0 + r^0_1 + a_2 b_0 + a_0 b_2 \end{aligned}$$

Unfortunately, Belaïd’s optimization cannot be generalized to higher orders to the best of our knowledge. As a second optimization we thus consider the DOM approach for handling this block which is again generic. DOM requires one addition less for the last $\varvec{q}$ function for $d=2$ but requires one random bit more than the Belaïd’s optimization (see Equation 6) and thus the same amount as Barthe et al.’s original algorithm. However, for the hardware implementation in the next sections the DOM approach saves area in this case because it can be parallelized.

6 $$\begin{aligned} q_0&= a_0 b_0 + r^0_0 + a_0 b_1 + r^0_2 + a_0 b_2 \nonumber \\ q_1&= a_1 b_1 + r^0_1 + a_1 b_2 + r^0_0 + a_1 b_0 \nonumber \\ q_2&= a_2 b_2 + r^0_2 + a_2 b_0 + r^0_1 + a_2 b_1 \end{aligned}$$

*Incomplete* Incomplete sequences contain only one multiplication term per share of $\varvec{q}$. In this case, a term is no longer added to its mirrored term. Instead, the association of each term with the shares of $\varvec{q}$ and the usage of the fresh random bits is performed according to the DOM scheme. An example for $d=1$ is given in Equation 7.

7 $$\begin{aligned} q_0&= a_0 b_0 + r^0_0 + a_0 b_1 \nonumber \\ q_1&= a_1 b_1 + r^0_0 + a_1 b_0 \end{aligned}$$

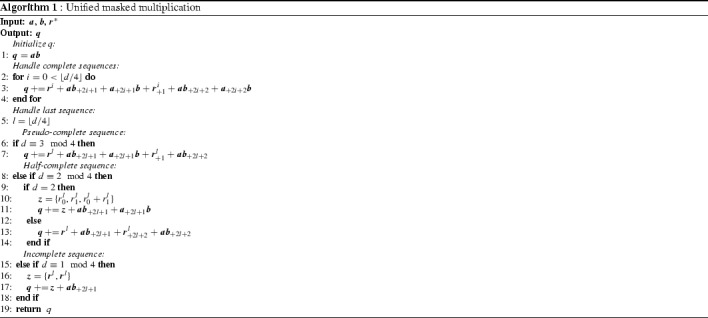


### Full description of UMA

Algorithm 1 shows the pseudocode of the proposed UMA algorithm. The inputs of the algorithm are the two operands $\varvec{a}$ and $\varvec{b}$ split into $d+1$ shares each. The randomness vector $\varvec{r}^*$ (we use $*$ to make it explicit that $\varvec{r}$ is a vector of vectors) contains $\lceil d/4 \rceil$ vectors with $d+1$ random bits each. Please note that all operations, including the multiplication and the addition, are again performed share-wise from left to right.

At first, the return vector *q* is initialized with the multiplication terms that have the same share index for *a* and *b* in Line 1. In Line 2 to 4, the *complete* sequences are calculated according to Barthe et al.’s original algorithm. We use the superscript indices to address specific vectors of $r^*$ and use again subscript indices for indexing operations on the vector. Subscript indices with a leading “$+$” denote a rotation by the given offset.

From Line 5 to 17 the handling of the remaining multiplication terms is performed according to the description above for the *pseudo-complete*, *half-complete*, and *incomplete* cases. In order to write this algorithm in quite compact form, we made the assumption that for the last random bit vector $\varvec{r}^l$ only the required random bits are provided. In Line 10 where Belaïd’s optimization is used for $d=2$, a new bit vector $\varvec{z}$ is formed that consists of the concatenation of the two elements of vector $\varvec{r^l}$ and the sum of these bits. So, in total the $\varvec{z}$ vector is again $d+1$ (three) bits long. In a similar way, we handle the randomness in Line 16. We concatenate two copies of $\varvec{r}^l$ of the length $(d+1)/2$ to form $\varvec{z}$ which is then added to the remaining multiplication terms.

*Randomness requirements* Table 1 shows a comparison of the randomness requirements of UMA with other masked multiplication algorithms. The comparison shows that UMA requires the least amount of fresh randomness in all generic cases. With the non-generic optimization by Belaïd et al., the algorithm reaches lower bounds of $d + 1$ for $d>2$ and of *d* for $d \le 2$ below the fifth protection order.

Compared to Barthe et al.’s original algorithm, UMA saves random bits in the cases where the last sequence is *incomplete*. More importantly, since we target efficient higher-order masked hardware implementations in the next sections, UMA has much lower randomness requirements than the so far most randomness efficient hardware masking scheme DOM. Up to half of the randomness costs can thus be saved compared to DOM. In the next section we show how UMA can be securely and efficiently implemented in hardware.

**Table 1:** Randomness requirement comparison

*d*	**UMA**	Barthe et al.	Belaïd et al.	DOM
1	**1**	2	**1**	**1**
2	3 (**2**$^\mathrm{a}$)	3	3 ($\mathbf 2 ^{1}$)	3
3	**4**	**4**	5 ($\mathbf 4 ^{1}$)	6
4	**5**	**5**	8 ($\mathbf 5 ^{1}$)	10
5	**9**	12	11	15
6	**14**	**14**	15	21
7	**16**	**16**	19	28
8	**18**	**18**	24	36
9	**25**	30	29	45
10	**33**	**33**	35	55
11	**36**	**36**	41	66
12	**39**	**39**	48	78
13	**49**	56	55	91
14	**60**	**60**	63	105
15	**64**	**64**	71	120

Bold denotes minimum of each category

$^\mathrm{a}$ non-generic solution

## UMA in hardware

Directly porting UMA to hardware by emulating what a processor would do, *i.e. * ensuring the correct order of instruction execution by using registers in between every operation, would introduce a tremendous area and performance overhead over existing hardware masking approaches. To make this algorithm more efficient while still keeping it secure in hardware, it needs to be sliced into smaller portions of independent code parts than can be translated to hardware modules which can be evaluated in parallel.

*Domain-oriented masking (DOM)* To discuss the security of the introduced hardware modules in the presence of glitches, we use the same terminology as DOM [14] in the following. DOM interprets the sharing of any function in hardware as a partitioning of the circuit into $d+1$ independent subcircuits which are also called domains. All shares of one variable are then associated with one specific domain according to their share index number ($a_0$ is associated with domain “0”, $a_1$ with domain “1”, et cetera.). By keeping the $d+1$ shares in their respective domains, the whole circuit is trivially secure against an attacker with the ability to probe *d* signals as required.

This approach is intuitively simple for linear functions that can be performed on each of the shares independently. To realize nonlinear functions, shared information from one domain needs to be sent to another domain in a secure way. This process requires the usage of fresh randomness without giving the attacker any advantage in probing all shares of any sensitive variable.

In the context of DOM, multiplication terms with the same share index (*e.g.* $a_0 b_0$) are also called *inner-domain* terms. These terms and are considered uncritical since the combination of information inside one domain can never reveal two or more shares of one variable as the domain itself contains only one share per variable. Terms which consist of shares with different share index (*cross-domain* terms) that thus originate from different domains (*e.g.* $a_0 b_1$) are considered to be more critical. Special care needs to be taken to ensure that at no point in time, *e.g. * due to timing effects (glitches), any two shares of one variable come together without a secure remasking step with fresh randomness in between.

**Fig. 2:**
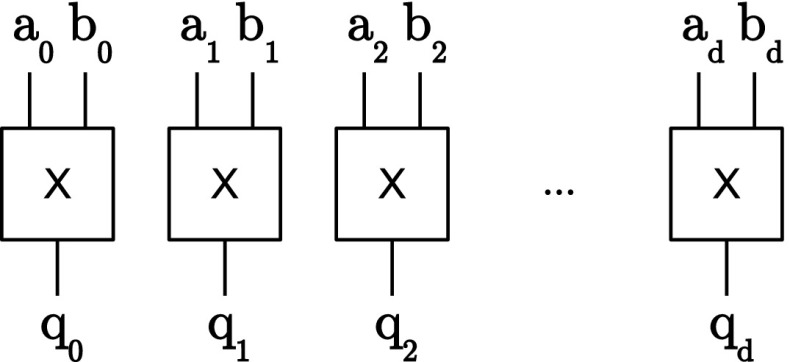
Inner-domain block

*Inner-domain block* The assignment of the inner-domain terms ($\varvec{q} = \varvec{a} \varvec{b}$) in Line 1 of Algorithm 1 can thus be considered uncritical in terms of *d*th-order probing security. Only shares with the same share index are multiplied and stored at the same index position of the share in $\varvec{q}$. The *inner-domain* block is depicted in Figure 2 and consists of $d+1$ AND gates that are evaluated in parallel. Hence, each share stays in its respective share domain. So even if the sharings of the inputs of *a* and *b* would be the same, this block does not provide a potential breach of the security because neither $a_0 a_0$ nor $b_0 b_0$, for example, would provide any additional information on *a* or *b*. We can thus combine the *inner-domain* block freely with any other secure masked component that ensures the same domain separation.

*(Pseudo-)complete blocks* For the security of the implementation in hardware, the order in which the operations in Line 3 (and Line 7) are performed is essential. Since the calculation of one *complete* sequence is subdivided by the addition of the random vector in the middle of this code line, it is quite tempting to split this calculation into two parts and to parallelize them to speed up the calculation.

However, if we consider Equation 4, and omit the inner-domain terms that would be already calculated in a separate inner-domain block, a probing attacker could get (through glitches) the intermediate results from the probe $p_0 = r_0 + a_0 b_1 + a_1 b_0$ from the calculation of $q_0$ and $p_1 = r_0 + a_4 b_1 + a_1 b_4$ from the calculation of $q_4$. By combining the probed information from $p_0$ and $p_1$ the attacker already gains information on three shares of *a* and *b*. With the remaining two probes the attacker could just probe the missing shares of *a* or *b* to fully reconstruct them. The *complete* sequence and for the same reasons also the *pseudo-complete* sequence can thus not be further parallelized.

**Fig. 3:**
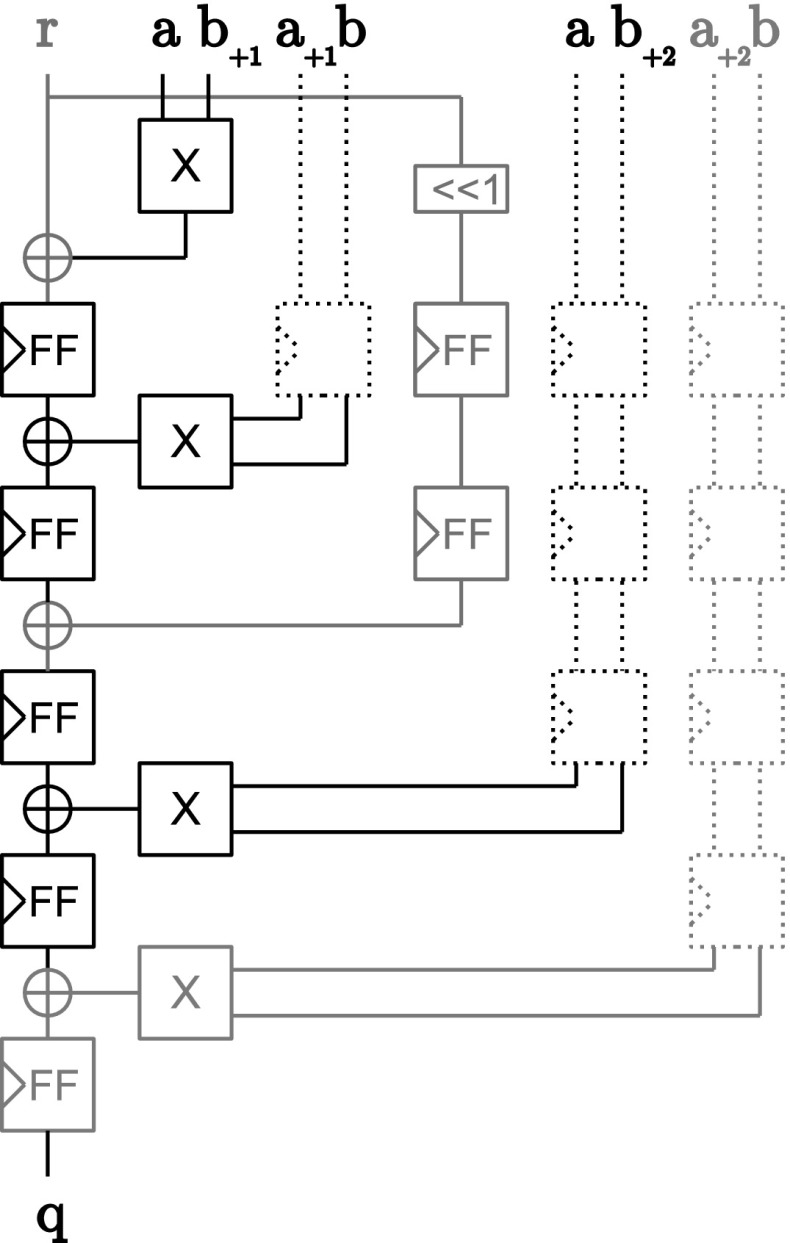
(Pseudo-)complete block

Figure 3 shows the vectorized *complete* block that consists of five register stages. Optional pipeline registers are depicted with dotted lines where necessary that make the construction more efficient in terms of throughput. For the *pseudo-complete* block, the last XOR is removed and the most right multiplier including the pipeline registers before the multiplier (marked green).

The security of this construction has already been analyzed by Barthe et al. [1] in conjunction with the inner-domain terms (which have no influence on the probing security) and for subsequent calculation of the sequences. Since the scope of the randomness vector is limited to one block only, a probing attacker does not gain any advantage (information on more shares than probes she uses) by combining intermediate results of different blocks even if they are calculated in parallel. Furthermore, each output of these blocks is independently and freshly masked and separated in $d+1$ domains which allows the combination with other blocks.

**Fig. 4:**
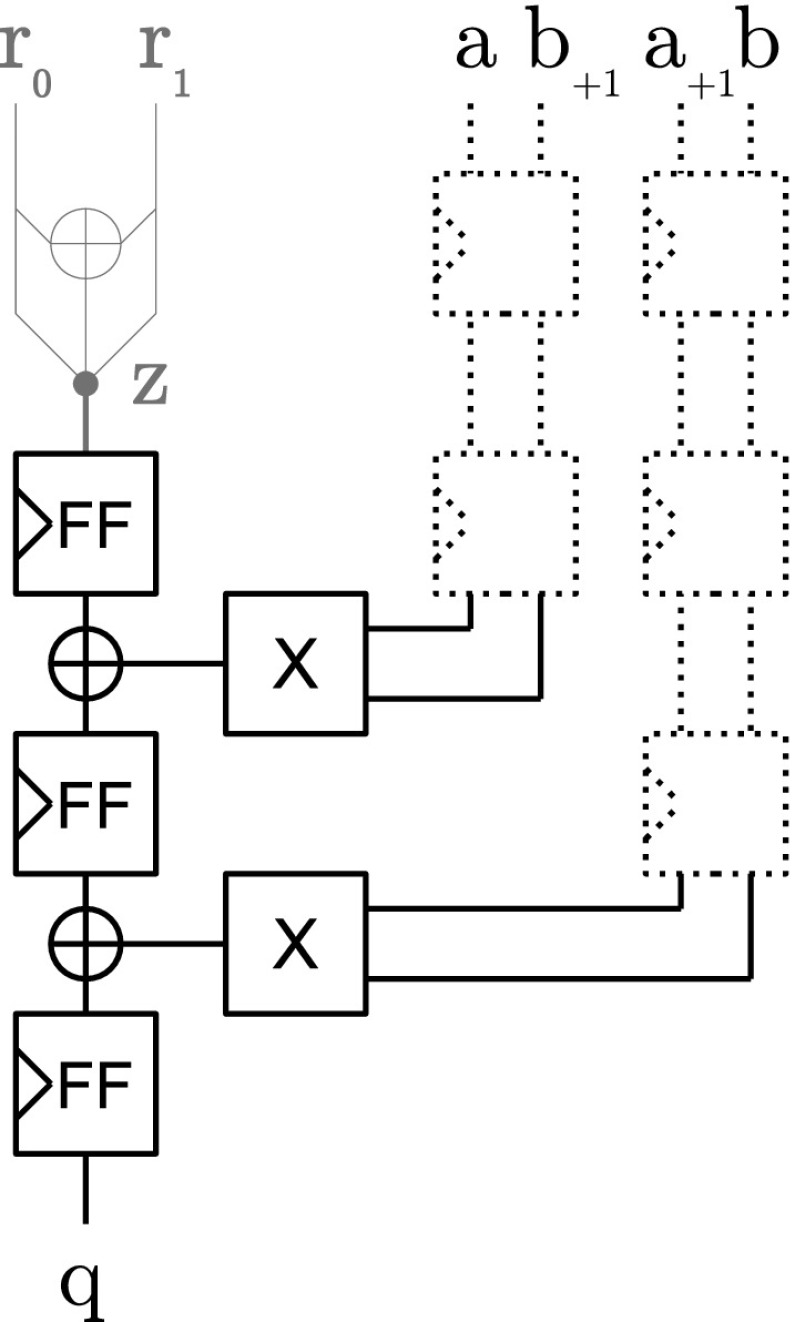
Half-complete block (Belaïd’s opt.)

*Half-complete block* Figure 4 shows the construction of the *half-complete* sequence in hardware when Belaïd’s optimization is used for $d=2$. The creation of the random vector $\varvec{z}$ requires one register and one XOR gate. The security of this construction was formally proven by Belaïd et al. in [2]. For protection orders other than $d=2$, we use instead the same DOM construction as we use for the incomplete block.

**Fig. 5:**
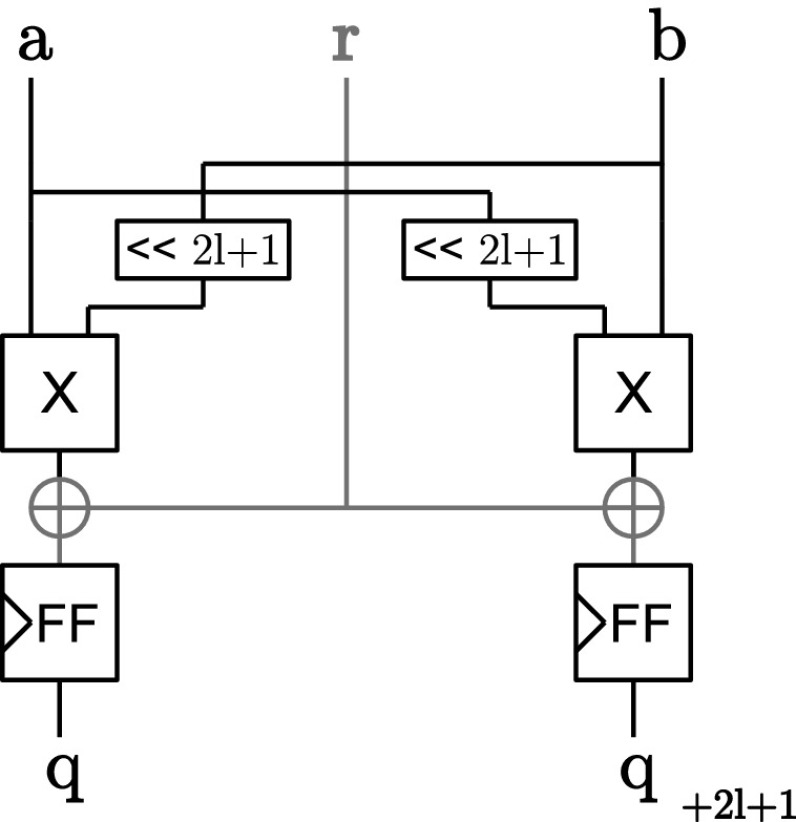
Incomplete block

*Incomplete block* For the *incomplete* block (and the half-complete block without Belaïd optimization) each random bit is only used to protect one multiplication term and its mirrored term. The term and the mirrored term are distributed in different domains to guarantee probing security. Figure 5 shows the construction of an *incomplete* block following the construction principles of DOM for two bits of $\varvec{q}$ at the same time. For *half-complete* blocks (without Belaïd’s optimization), two instances of the *incomplete* constructions are used with different indexing offsets and the resulting bits are added together (see Line 13). No further registers are required for the XOR gate at the output of this construction because it is ensured by the registers that all multiplication terms are remasked by *r* before the results are added. For a more detailed security discussion we refer to the original paper of Gross *et al.* [14].

**Fig. 6:**
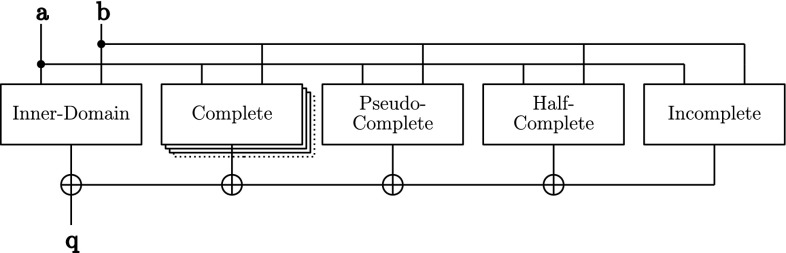
Fully assembled UMA AND gate

*Assembling the UMA AND gate* Figure 6 shows how the UMA AND gate is composed from the aforementioned building blocks. Except for the *inner-domain* block which is always used, all other blocks are instantiated and connected depending on the given protection order which allows for a generic construction of the masked AND gate from $d=0$ (no protection) to any desired protection order. Connected to the *inner-domain* block, there are $\lfloor \frac{d}{4} \rfloor$ *complete* blocks, and either one or none of the *pseudo-complete*, *half-complete*, or *incomplete* blocks.

Table 2 gives an overview of the hardware costs of the different blocks that form the masked AND gate. All stated gate counts need to be multiplied by the number of shares ($d+1$). The XOR gates which are required for connecting the different blocks are accounted to the *inner-domain* block. In case pipelining is used, the input shares of *a* and *b* are pipelined instead of pipelining the multiplication results inside the respective blocks. The required pipelining registers for the input shares are also added to the *inner-domain* block’s register requirements, since this is the only fixed block of every masked AND gate. The number of pipelining registers is determined by the biggest delay required for one block. In case one or more *complete* blocks are instantiated, there are always five register stages required which gives a total amount of $10 (d+1)$ input pipelining registers. However, for $d<4$ the number of input pipelining registers is always twice the amount of latency cycles of the instantiated block which could also be zero for the unprotected case where the masked AND gate consists only of the *inner-domain* block. The *inner-domain* block itself does not require any registers except for the pipelining case and thus has a latency of zero.

**Table 2:** Overview of the hardware costs of the different UMA AND blocks (gate counts need to be multiplied by $d+1$)

Block	AND	XOR	FF		Delay
				(Pipel.)	
Inner-domain	1	$\lceil \frac{d}{4} \rceil$	0	$(0-10)$	0
Complete	4	5	5	(7)	5
Pseudo-complete	3	4	4	(6)	4
Half-complete					
Belaïd’s opt.	2	$2\frac{1}{3}$	3	(3)	3
DOM	2	3	2	(2)	1
Incomplete	1	1	1	(1)	1

For the cost calculation of the UMA AND gate, the gate counts for the *complete* block need to be multiplied by the number of instantiated *complete* blocks ($\lfloor \frac{d}{4} \rfloor$) and the number of shares ($d+1$). The other blocks are instantiated at maximum once. The *pseudo-complete* block in case $d \equiv 3 \mod 4$, the *half-complete* block in case $d \equiv 2 \mod 4$ (where Belaïd’s optimization is only used for $d=2$), and the *incomplete* block in case $d \equiv 1 \mod 4$.

**Table 3:** Comparison of the UMA AND gate with DOM

*d*	UMA AND						DOM AND					
	AND	XOR	Registers		GE		AND	XOR	Registers		GE	
			Unpipel.	Pipel.	Unpipel.	Pipel.			Unpipel.	Pipel.	Unpipel.	Pipel.
1	4	4	2	6	24	42	4	4	2	4	24	33
2	9	10	9	27	77	157	9	12	6	9	68	82
3	16	20	16	56	142	322	16	24	12	16	134	152
4	25	30	25	85	219	489	25	40	20	25	221	244
5	36	48	36	108	327	651	36	60	30	36	330	357
6	49	70	49	133	457	835	49	84	42	49	460	492
7	64	88	72	184	624	1128	64	112	56	64	612	648
8	81	108	90	216	776	1343	81	144	72	81	785	826
9	100	140	110	250	970	1600	100	180	90	100	980	1025
10	121	176	132	286	1185	1878	121	220	110	121	1196	1246
11	144	204	168	360	1446	2310	144	264	132	144	1434	1488
12	169	234	195	403	1674	2610	169	312	156	169	1693	1752
13	196	280	224	448	1953	2961	196	364	182	196	1974	2037
14	225	330	270	510	2321	3401	225	420	210	225	2276	2344
15	256	368	304	592	2608	3904	256	480	240	256	2600	2672

**Fig. 7:**
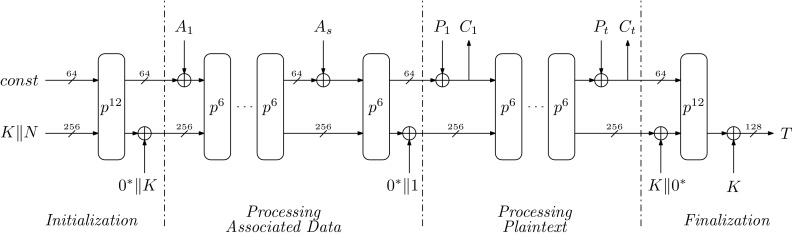
Data encryption and authentication with Ascon, from [8]

*Comparison with DOM* Table 3 shows a first comparison of the UMA AND gate with a masked AND gate from the DOM scheme. For the generation of these numbers we used Table 2 to calculate the gate counts for the UMA AND gate. For DOM, we used the description in [14] which gives us $(d+1)^2$ AND gates, $2 d (d+1)$ XOR gates, and $(d+1)^2$ registers (${}-d-1$, for the unpipelined variant). For calculating the gate equivalence, we used the 90 nm UMC library from Faraday as reference as we also use them for synthesis in Sect. 5. Accordingly, a two input AND gate requires 1.25 GE, an XOR gate 2.5 GE, and a D-type flip-flop with asynchronous reset 4.5 GE.

Since in both implementations AND gates are only used for creating the multiplication terms, both columns for the UMA AND gate construction and the DOM AND are equivalent. The gate count for the XORs in our implementation is lower than for the DOM gate which results from the reduced randomness usage compared to DOM. The reduced XOR count almost compensates for the higher register usage in the unpipelined case. The difference for the 15th order is still only 8 GE, for example. However, the latency of the UMA AND gate is in contrast to the DOM AND gate, except for $d=1$, not always one cycle but increases up to five cycles. Therefore, in the pipelined implementation more registers are necessary which results in an increasing difference in the required chip area for higher protection orders.

## Case study on Ascon

To show the suitability of the UMA approach and to study the implications on a practical design, we decide on implementing the CAESAR candidate Ascon [8] one time with DOM and one time with the UMA approach. We decided on Ascon over the AES for example, because of its relatively compact S-box construction which allows to compare DOM versus UMA for a small percentage of nonlinear functionality, but also for a high percentage of nonlinear functionality if the S-box is instantiated multiple times in parallel. The design is for both DOM and UMA generic in terms of protection order and allows some further adjustments. Besides the different configuration parameters for the algorithm itself, like block sizes and round numbers, the design also allows to set the number of parallel S-boxes and how the affine transformation in the S-box is handled, for example.

**Fig. 8:**
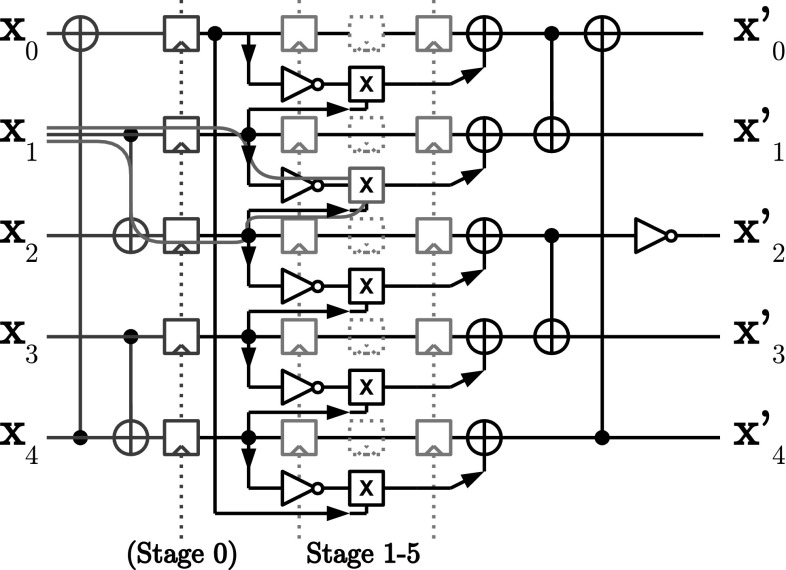
Overview of the Ascon core (left) and the state module (right)

Ascon is an authenticated encryption scheme with a sponge-like mode of operation as depicted in Figure 7. The encryption and decryption work quite similar. At the beginning of the initialization the 320-bit state is filled with some cipher constants, the 128-bit key *K*, and the 128-bit nonce *N*. In the upcoming calculation steps, the state performs multiple rounds of the transformation *p* which consists of three substeps: (1) the addition of the round constant, (2) the nonlinear substitution layer, and (3) the linear transformation. For the Ascon-128 the initialization and the finalization takes 12 rounds and the processing of any data takes six rounds. The input data are subdivided into associated data (data that only require authentication but no confidentiality) and plaintext or ciphertext data. The data are processed by absorbing the data in 64-bit chunks into the state and subsequently performing the state transformation. In the finalization step, a so-called tag is either produced or verified that ensures the authenticity of the processed data.

### Proposed hardware design

An overview of the top module of our hardware design is given in Figure 8 (left). It consists of a simple data interface to transfer associated data, plaintext or ciphertext data with ready and busy signaling which allows for simple connection with *e.g. * AXI4 streaming masters. Since the nonce input and the tag output have a width of 128 bit, they are transferred via a separate port. The assumptions taken on the key storage and the random number generator (RNG) are also depicted. We assume a secure key storage that directly transfers the key to the cipher core in shared form, and an RNG that has the capability to deliver as many fresh random bits as required by the selected configuration of the core.

The core itself consists of the *control FSM* and the *round counter* that form the control path, and the *state* module that forms the data path and is responsible for all state transformations. Figure 8 (right) shows a simplistic schematic of the state module. The state module has a separate FSM and performs the round transformation in four substeps:

1. during *IDLE*, the initialization of the state with the configuration constants, the key, and the nonce is ensured.
2. in the *ROUND_CONST* state the round constant is added, and optionally other required data are either written or added to the state registers like input data or the key. Furthermore, it is possible to perform the linear parts of the S-box transformation already in this state to save pipeline registers during the S-box transformation and to save one cycle. This option, however, is only used for the configuration of Ascon where all 64 possible S-box instances are instantiated.
3. the *SBOX_LAYER* state provides flexible handling of the S-box calculation with a configurable number of parallel S-box instances. Since the S-box is the only nonlinear part of the transformation, its size grows quadratically with the protection order and not linearly as the other data path parts of the design. The configurable number of S-boxes thus allows to choose a trade-off between throughput and chip area, power consumption, et cetera. During the S-box calculation, the state registers are shifted and the S-box module is fed with the configured number of state slices with five bits each slice. The result of the S-box calculation is written back during the state shifting. Since the minimum latency of the S-box changes with the protection order and whether the DOM or UMA approach is used, the S-box calculation takes one to 70 cycles.
4. in the *LINEAR_LAYER* state the whole linear part of the round transformation is calculated in a single clock cycle. The linear transformation simply adds two rotated copies of one state row with itself. It would be possible to breakdown this step into smaller chunks to save area. However, the performance overhead and the additional registers required to do so, would relativize the chip area savings especially for higher orders.

**Fig. 9:**
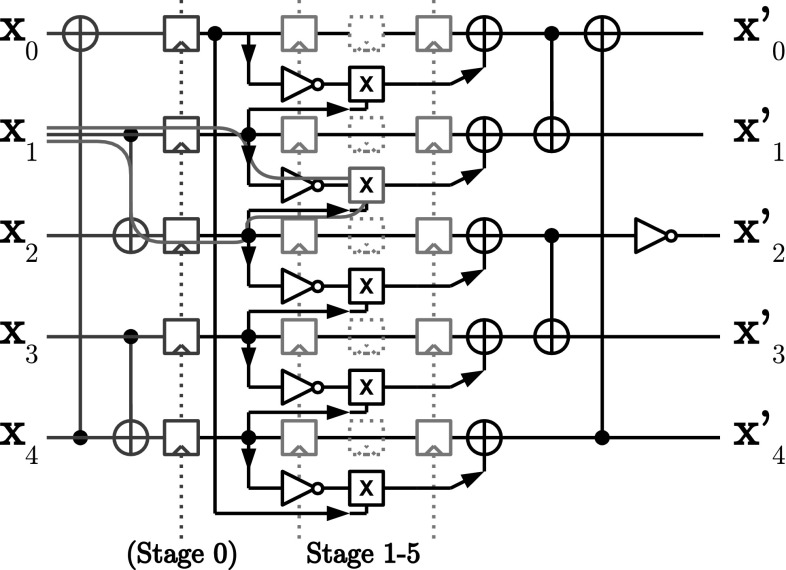
Ascon’s S-box module with optional affine transformation at input (gray) and variable number of pipeline registers (green)

*S-box construction* Ascons’s S-box is affine equivalent to the Keccak S-box and takes five (shared) bits as an input (see Figure 9).

**Fig. 10:**
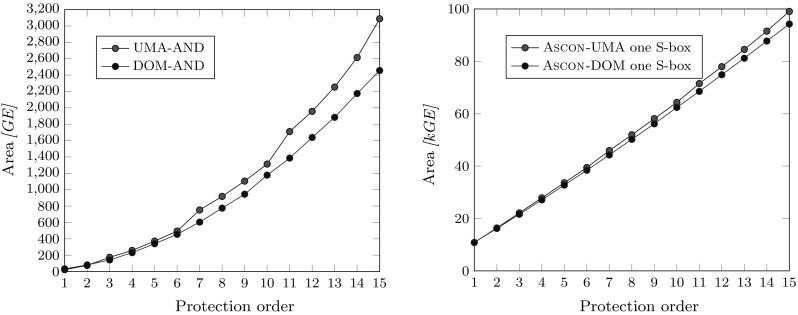
UMA versus DOM area requirements for different protection orders. Left figure compares masked AND gates, right figure compares full Ascon implementations

The figure shows where the pipeline registers are placed in our S-box design (green dotted lines). The first pipeline stage (Stage 0, gray) is optionally already calculated in the *ROUND_CONST* stage. The registers after the XOR gate in State 0 are important for the glitch resistance and therefore for the security of the design. Without this registers, the second masked AND gate from the top (red paths), for example, could temporarily be sourced two times by the shares of $x_1$ for both inputs of the masked AND gate. Because the masked AND gate mixes shares from different domains, a timing dependent violation (glitch) of the *d*-probing resistance could occur. Note that the XOR gates at the output do not require an additional register stage because they are fed into one of the state registers. As long as no share domains are crossed during the linear parts of the transformation the probing security is thus given. We assure this by associating each share and each part of the circuit with one specific share domain (or index) and keeping this for the entire circuit.

The other pipelining registers are required because of the latency of the masked AND gates which is one cycle for the DOM gate, and up to five cycles for the UMA AND gate according to Table 2.

### Implementation results

All results stated in this section are post-synthesis results for a 90 nm Low-K UMC process with 1 V supply voltage and a 20 MHz clock. The designs were synthesized with the Cadence Encounter RTL compiler v14.20-s064-1. Figure 10 compares the area requirements of the UMA approach with DOM for the pipelined Ascon implementation with a single S-box instance. The figure on the left shows the comparison of single masked AND gates inside the Ascon design, while the figure on the right compares the whole implementations of the design. Comparing these results with Table 3 reveals that the expected gate counts for DOM match the practical results quite nicely. For the UMA approach, on the other hand, the practical results are always lower than the stated numbers. The reduction results from the fact that the amount of required pipelining registers for the operands is reduced because the pipelining register is shared among the masked AND gates. This does not affect the DOM implementation because the multiplication results are always calculated within only one cycle.

**Fig. 11:**
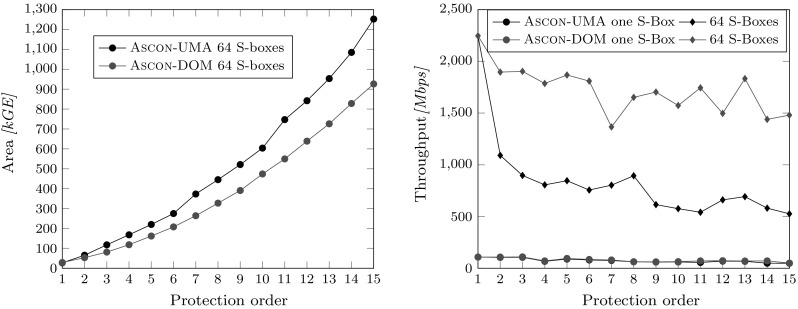
UMA versus DOM area requirements for different protection orders with 64 parallel S-boxes (left) and throughput comparison in the right figure

The right figure shows that the difference for the single S-box Ascon implementation is relatively low especially for low protection orders, and seems to grow only linearly within the synthesized range for *d* between 1 and 15. For the first-order implementation both designs require about 10.8 kGE. For the second-order implementation the difference is still only about 200 GE (16.2 kGE for DOM versus 16.4 kGE). The difference grows with the protection order and is about 4.8 kGE for $d=15$ which is a size difference of about 5 %. The seemingly linear growth in area requirements for both approaches is observed because the S-box is only a relatively small part with 3–20 % of the design which grows quadratically, while the state registers that grow linearly dominate the area requirements with 96–80 %.

We also synthesized the design for 64 parallel S-boxes which makes the implementation much faster in terms of throughput but also has a huge impact on the area requirements (see Figure 11). The characteristics for UMA and DOM look quite similar to the comparison of the masked AND gates in Figure 10 (left) and shows a quadratic increase with the protection order. The chip area is now between 28 kGE ($d=1$) and 1,250 kGE ($d=15$) for UMA and 926 kGE for DOM. The S-box requires between 55 % and 92 % of the whole chip area.

*Throughput* To compare the maximum throughput achieved by our designs we calculated the maximum clock frequency for which our design is expected to work for typical operating conditions (1 V supply, and $25\,^\circ \hbox {C}$) over the timing slack for the longest delay path. This frequency is then multiplied with the block size for our encryption (64 bits) divided by the required cycles for absorbing the data in the state of Ascon (for six consecutive round transformations).

The results are shown in Figure 11. The throughput of both masking approaches with only one S-box instance is quite similar which can be explained with the high number of cycles required for calculating one-round transformation (402-426 cycles for UMA versus 402 cycles for DOM). The UMA approach achieves a throughput between 48 Mbps and 108 Mbps, and the DOM design between 50 Mbps and 108 Mbps for the single S-box variants.

For 64 parallel S-boxes, the gap between DOM and UMA increases because DOM requires only 18 cycles to absorb one block of data while UMA requires between 18 and 42 cycles which is an overhead of more than 130 %. Therefore, also the throughput is in average more than halved for the UMA implementation. The UMA design achieves between 0.5 Gbps and 2.3 Gbps, and DOM Ascon between 1.5 Gbps and 2.3 Gbps.

*Randomness* The amount of randomness required for the UMA and DOM designs can be calculated from Table 1 by multiplying the stated number by five (for the five S-box bits), and additionally by 64 in case of the 64 parallel S-box version. For the single S-box design, the (maximum) amount of randomness required per cycle for the UMA design is thus between 5 bits for $d=1$ and 320 bits for $d=15$, and for DOM between 5 bits and 600 bits. For the 64 parallel S-boxes design, the first-order designs already require 320 bits per cycle, and for the 15th-order designs the randomness requirements grow to 20 kbits and 37.5 kbits per cycle, respectively.

## Empirical side-channel evaluation

In order to analyze the side-channel analysis resistance of our implementations, we performed a statistical *t* test according to Goodwill et al. [10] on leakage traces of the S-box designs of the UMA variants. We note that *t* tests are unfeasible to prove any general statements on the security of a design (for all possible conditions and signal timings) as it would be required for a complete security verification. *T* tests only allow statements for the tested devices and under the limitations of the measurement setup. Many works test masked circuits on an FPGA and perform the *t* test on the traces gathered from power measurements. This approach has the drawback that due to the relatively high noise levels the evaluation is usually limited to first- and second-order multivariate *t* tests.

However, in practice *t* tests have proven to be very sensitive and useful to test the side-channel resistance of a design. So we utilize *t* testing to increase the confidence in the correctness and security of our implementations. A formal analysis of the S-box construction for UMA is then given in the next section.

**Fig. 12:**
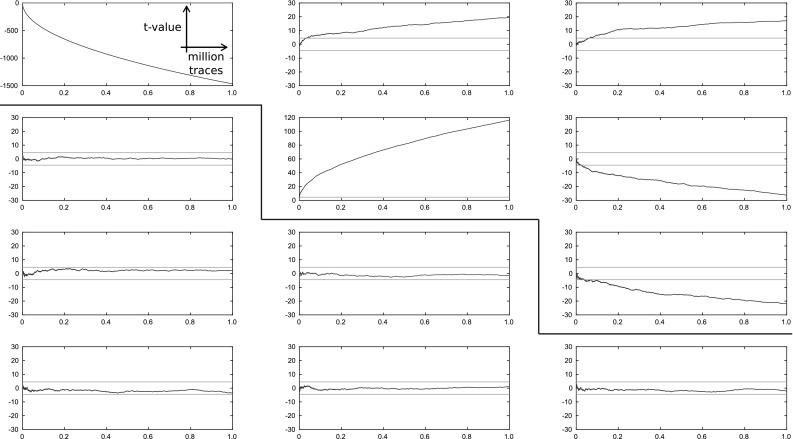
*T* test evaluation for different protection orders $d=0\dots 3$ (from top to bottom) and for different *t* test orders (first to third, from left to right)

*Setup* We use the recorded signal traces from the post-synthesis simulations which are noise-free and allows us to evaluate the designs up to the third order. Using post-synthesis leakage traces over traces collected from an FPGA or ASIC design shows some differences which are in some case very beneficial, but there are also drawbacks. First of all, post-synthesis leakage traces are totally free from environmental noise and variations in the operating conditions like temperature or the supply voltage. As a result, violations of the *d*th-order security are found with much less leakage traces. Another big advantage is that the *t* tests can be performed either on a rather coarse level, by taking all signals together into account, or on a very fine-grained level by using individual signals. Latter one allows to directly locate the source of the leakage on signal level which makes it very easy to find the flaws in the design.

One disadvantage is that post-synthesis traces do not use a real existent leakage source. A *t* test performed on a specific ASIC chip or FPGA design, however, also only allows to give a statement about this specific device and even does not give a guarantee about its behavior in the future, since signal delays may change under different environmental conditions and over the life cycle of a device. In case of the simulated synthesized netlist, the signal delays are based on unified gate delays which also result in signal glitches that appear from cascading logic gates. Glitches that would result from different wire lengths, and other parasitic effect, however, are not modeled and thus are more likely to show up on FPGA or ASIC based *t* tests.

The intuition of the *t* test follows the idea that an DPA attacker can only make use of differences in leakage traces. To test that a device shows no exploitable differences, two sets of traces are collected per *t* test: (1) a set with randomly picked inputs, (2) a set with fixed inputs and the according *t* value is calculated. Then the *t* value is calculated according to Equation 8 where *X* denotes the mean of the respective trace set, $S^2$ is the variance, and *N* is the size of the set, respectively.

8 $$\begin{aligned} t = {{{X_1} - {X_2}}\over {\sqrt{{{S^2_1}\over {N_1}} + {{S^2_2}\over {N_2}}}}} \end{aligned}$$

The null-hypothesis is that the means of both trace sets are equal, which is accepted if the calculated *t* value is below the border of $\pm \,4.5$. If the *t* value exceeds this border then the null-hypothesis is rejected with a confidence greater than 99.999% for large enough trace sets. A so-called centered product preprocessing step with trace points inside a six cycle time window is performed for higher-order *t* tests. Beyond this time frame, the intermediates are ensured to be unrelated to the inputs. We thus combine multiple tracepoints by first normalizing the means of the trace points and then multiplying the resulting values with other normalized points inside the time window.

*Results* Figure 12 shows the results of the *t* tests for the time offsets which achieved the highest *t* values for the UMA S-box implementations of Ascon. From top to bottom the figures show the results for different protection orders from $d=0$ to $d=3$, and from left to right we performed different orders of *t* tests starting from first order up to third order. Above $d=3$ and third-order *t* tests the evaluation of the *t* tests becomes too time intensive for our setup.

On the *y*-axis of the figures the *t* values are drawn, and the *y*-axis denotes the used number of traces at a fraction of a million. The horizontal lines (green, inside the figures) indicate the $\pm \,4.5$ confidence border. The protection border between the figures (the red lines) separates the *t* tests for which the protection order of the design is below the performed *t* test (left) from the *t* tests for which the test order is above (right).

As intended, the *t* values for the masked implementations below the protection border do not show any significant differences even after one million noise-free traces. For the unprotected implementation (top, left figure), for example, the first-order *t* test fails with great confidence even after only a couple of traces, and so do the second- and third-order *t* tests on the right. The first-order *t* test below of the first-order protected S-box does not show leakages anymore, but the higher-order *t* tests fail again as expected. The third-order implementation does not show any leakages anymore for the performed *t* tests. We thus conclude that our implementations seem to be secure under the stated limitations.

## Formal side-channel verification

In this section, we strengthen the side-channel evaluation of the UMA S-box construction by an additional formal verification according to Bloem et al. [4]. In contrast to empirical verification approaches, like the *t* tests in Sect. 6, the security statement is valid beyond the used measurement method, the number of used traces, the actual signal transition times, the concrete verified device (FPGA or ASIC chip), the environmental conditions, et cetera. Furthermore, this formal approach allows to pinpoint a detected flaw directly on the circuits netlist. We used the Z3 theorem solver instance of the verification approach (which is available online [17]) to check for possible leakages in the S-box construction for UMA.

The main idea of this formal verification tool is the calculation of the Fourier expansion of a circuit for all possible signal timings which therefore also covers glitches. The Fourier expansion (or Walsh transformation) of a circuit allows to determine which signal components leak by probing a certain wire. To calculate the Fourier spectrum *e.g. * of a simple AND gate (leaving the signal propagation time aside), the Boolean values *true* and *false* are first mapped to $-1$ and 1, respectively. The Fourier expansion can then be calculated according to Definition 1 over the function of an AND gate.

### Definition 1

(*Fourier expansion according to* [4]) A Boolean function $f: \{-1, 1\}^n \rightarrow \{ -1, 1 \}$ can be *uniquely* expressed as a multilinear polynomial in the *n*-tuple of variables $X = (x_1, x_2, \dots , x_n)$ with $x_i\in \{\pm \,1\}$, *i.e. * the multilinear polynomial of *f* is a linear combination of monomials, called *Fourier characters*, of the form $\chi _T(X) = \prod _{x_i\in T}x_i$ for every subset $T \subseteq X$. The coefficient of $\chi _T \in \mathbb {Q}$ is called the *Fourier coefficient* $\widehat{f}(T)$ of the subset *T*. Thus, we have the *Fourier representation* of *f*:

$$\begin{aligned} f(X)=\sum _{T \subseteq X}\widehat{f}(T) \chi _T(X) = \sum _{T \subseteq X}\widehat{f}(T) \prod _{x_i\in T}x_i. \end{aligned}$$

In other words, a function like $f(x,y) = x \wedge y$ can be written as the sum over all possible combinations of input variables ($\emptyset$, *x*, *y*, $x \cdot y$) multiplied by the respective Fourier coefficients:

$$\begin{aligned} 1/2 + 1/2 \cdot x + 1/2 \cdot y - 1/2 \cdot x \cdot y \end{aligned}$$

This equation evaluates to *true* (-1) if and only if *x* and *y* are *true*, and otherwise *false* (1). The spectrum of the Fourier expansion (coefficients $\widehat{f}(T)$) determines which signals and signal combinations correlate with the function and thus have a nonzero coefficient. The AND function thus correlates with *x*, with *y*, and the combination of *x* and *y*, and is furthermore not uniformly distributed because the $\emptyset$ component has a nonzero coefficient.

Because the calculation of the precise Fourier spectrum of a circuit under all possible signal timings soon becomes unfeasible, the verification tool approximates the calculation of the spectrum by using a rule based system for the calculation (for more details we refer the interested reader to [4]). This set of rules ensure that the actual spectrum of the circuit is indeed part of the approximated spectrum.

The required inputs, despite the netlist, are the verification order *d*, and the class and dependencies of the port signals. There exist three classes which are denoted *S* for shared inputs or secrets, *M* for uniformly random masks, and *P* for uncritical or publicly known signals which can be ignored in the actual analysis (like the clock, round constants, but also outputs). For the first-order UMA S-box (available at [11]), for example, we used the labeling for the ports of the top module as shown in Table 4.

**Table 4:** Input labeling for the verification of the UMA S-box

Signal name(s)	Class	Description
X0xDI - X4xDI	S	Shared input signals
Z0xDI - Z4xDI	M	Fresh random bits
ClkxCI	P	Clock signal
RstxBI	P	Reset signal
X0xDO - X4xDO	P	Output signal

The verification results are shown in Table 5. We used the optimization introduced in [4] for which each shared input is checked individually. The time stated in the table is the average verification time over each of the five secret inputs. The first-order protected UMA S-box requires about one second per secret. The verification time increases significantly with the verification order. For the second order the verification takes about 1.5 min, and for the third order it is already about 20 h. Again all verification results indicate a secure design of the S-box which confirms the results of the *t* test.

**Table 5:** Formal verification results of the UMA S-box

Design	Verification order	Time	Result
1st-order S-box	1	$\le 1$ s	✓
2nd-order S-box	2	$\le 1.5$ m	✓
3rd-order S-box	3	$\le 20$ h	✓

## Discussion on the randomness costs and conclusions

In this work, we combined software and hardware-based masking approaches into a unified masking approach (UMA) in order to save randomness and the cost involved. In practice, the generation of fresh randomness with high entropy is a difficult and costly task. It is, however, also difficult to put precise numbers on the cost of randomness generation because there exist many possible realizations. The following comparison should thus not be seen as statement of implementation results but reflects only one possible realization which serves as basis for the discussion.

**Fig. 13:**
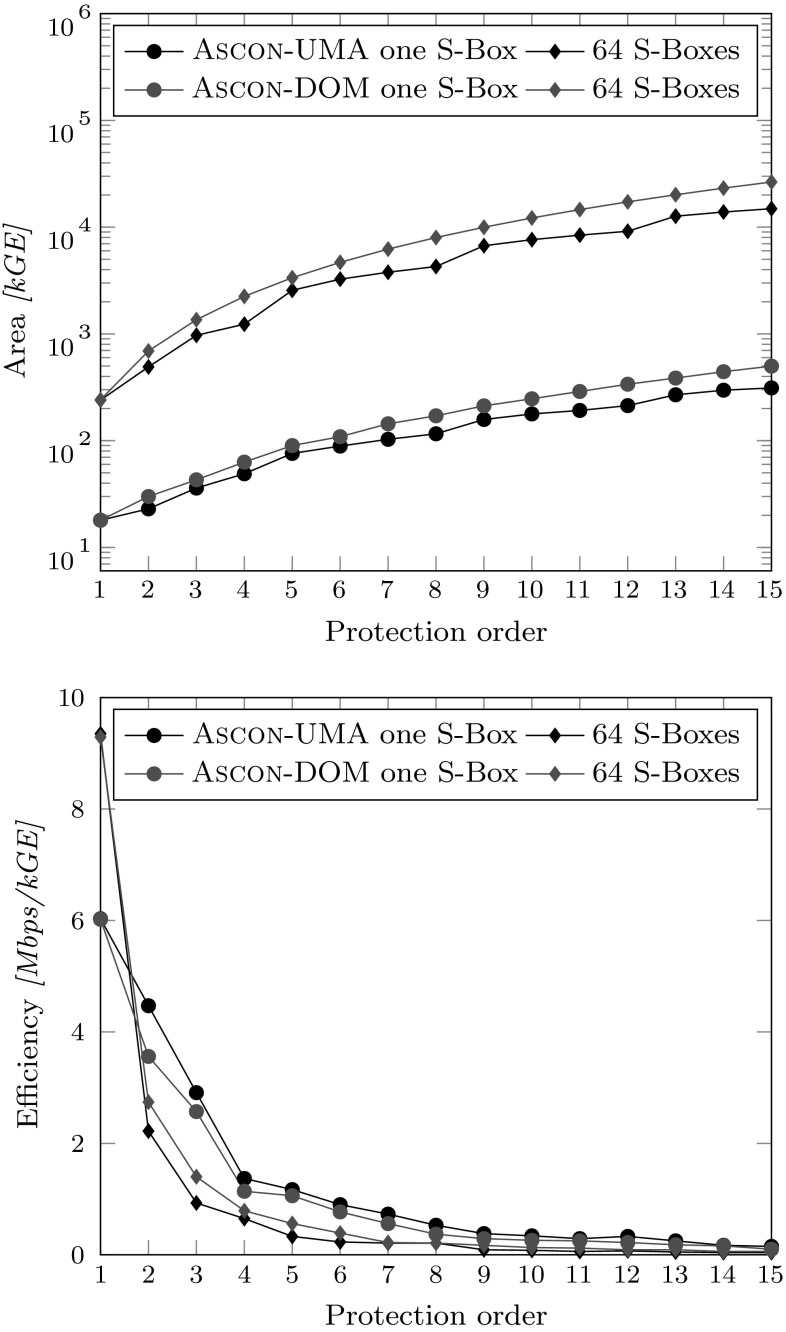
UMA versus DOM area requirements including an area estimation for the randomness generation in the top figure, and an efficiency evaluation (throughput per chip area) on the bottom

A common and performant way to generate many random numbers with high entropy is the usage of pseudo-random number generators (PRNGs) based on symmetric primitives, like Ascon for example. A single cipher design thus provides a fixed number of random bits, *e.g. * 64 bits in the case of Ascon, every few cycles. In the following comparison, we assume a one-round unrolled Ascon implementation resulting in six cycles latency and 7.1 kGE of chip area [15]. If more random bits are required, additional PRNGs are inserted, which increase the area overhead accordingly.

Figure 13 (top) shows the area results from Sect. 5 including the overhead cost for the required PRNGs. Starting with $d=2$ for DOM, $d=3$ for UMA for the single S-box variants, and for all of the 64 parallel S-box variants, one PRNG is no longer sufficient to reach the maximum possible throughput the designs offer. The randomness generation thus becomes the bottleneck of the design, and additional PRNGs are required, which result in the chip area differences compared to Figures 10 and 11, respectively. As depicted, both UMA variants require less chip area than their DOM pendants, but this comparison does not take the throughput of the designs into account (see Figure 11).

Figure 13 (bottom) compares the efficiency, calculated as throughput (in Mbps) over the chip area (in kGE). By using this metric, it shows that UMA is the more efficient scheme when considering the single S-box variants, while DOM is the more efficient solution for the 64 S-box variants. However, the practicality of the 64 S-box implementations with up to a few millions of GE and between 30 and 3,600 additional PRNGs is very questionable.

In practice, the most suitable approach for generating random bits and the constraints vary from application to application. While UMA seems to be the more suitable approach for low-area applications, DOM introduces less latency which is a relevant constraint for performance-oriented applications. To make our results comparable for future designs and under varying constraints, we make our hardware implementations available online [11].
